# A knowledge synthesis of culturally- and spiritually-sensitive end-of-life care: findings from a scoping review

**DOI:** 10.1186/s12877-016-0282-6

**Published:** 2016-05-18

**Authors:** Mei Lan Fang, Judith Sixsmith, Shane Sinclair, Glen Horst

**Affiliations:** Gerontology Research Centre, Simon Fraser University, 2800-515 West Hastings Street, Vancouver, BC V6B 5 K3 Canada; Institute of Health and Wellbeing, University of Northampton, Northampton, UK; School of Public Policy, Simon Fraser University, Vancouver, BC Canada; Faculty of Nursing, University of Calgary, Calgary, AB Canada; Hospice Clinical Team, Canadian Virtual Hospice, Winnipeg, MB Canada

**Keywords:** Terminal care, Cultural competency, Spirituality, Review, Ethnic groups, Health knowledge, Attitudes & practice

## Abstract

**Background:**

Multiple factors influence the end-of-life (EoL) care and experience of poor quality services by culturally- and spiritually-diverse groups. Access to EoL services e.g. health and social supports at home or in hospices is difficult for ethnic minorities compared to white European groups. A tool is required to empower patients and families to access culturally-safe care. This review was undertaken by the Canadian Virtual Hospice as a foundation for this tool.

**Methods:**

To explore attitudes, behaviours and patterns to utilization of EoL care by culturally and spiritually diverse groups and identify gaps in EoL care practice and delivery methods, a scoping review and thematic analysis of article content was conducted. Fourteen electronic databases and websites were searched between June–August 2014 to identify English-language peer-reviewed publications and grey literature (including reports and other online resources) published between 2004–2014.

**Results:**

The search identified barriers and enablers at the systems, community and personal/family levels. Primary barriers include: cultural differences between healthcare providers; persons approaching EoL and family members; under-utilization of culturally-sensitive models designed to improve EoL care; language barriers; lack of awareness of cultural and religious diversity issues; exclusion of families in the decision-making process; personal racial and religious discrimination; and lack of culturally-tailored EoL information to facilitate decision-making.

**Conclusions:**

This review highlights that most research has focused on decision-making. There were fewer studies exploring different cultural and spiritual experiences at the EoL and interventions to improve EoL care. Interventions evaluated were largely educational in nature rather than service oriented.

**Electronic supplementary material:**

The online version of this article (doi:10.1186/s12877-016-0282-6) contains supplementary material, which is available to authorized users.

## Background

End-of-life (EoL) care requires attention to psychological, social and spiritual needs and supports to help individuals cope with the process of aging, coming to terms with death and dying and to help family members and loved ones cope with bereavement [1]. Research reveals that access to EoL services such as health services and social supports either at home or in hospices are particularly low among ethnic minorities living in Western societies when compared to white populations [2, 3].

There are multiple factors that influence low-uptake of and lack of sensitivity in EoL care that contribute to poor quality service experiences of culturally- and spiritually-diverse groups [4]. For this work, a cultural group is defined in terms of their shared stories, beliefs, values, myths and practices shaped by history and geography. A spiritual group is characterized in terms of religious, spiritual and faith-based beliefs and practices. Key barriers to EoL care include: cultural differences between healthcare providers (HCPs) and persons approaching EoL, patients and families [5]; under-utilization of culturally-sensitive models designed for improved EoL care [6]; language barriers [7]; lack of awareness of cultural and spiritual diversity issues; exclusion of families in the decision-making process [8]; personal racial and religious discrimination [9] and lack of culturally-tailored EoL information to facilitate decision-making and uptake of care for culturally- and spiritually-diverse communities [9]. These factors indicate the centrality of Western bioethics whereby EoL decision-making and care delivery are shaped by spirituality, religion and culture as well as the beliefs of individuals, families, communities and other social structures within these groups. To understand how to improve health care for people inhabiting minoritized social positions, we need an understanding of how health care systems intersect with people’s experiences. This scoping review thus explores the notions of EoL care from both the systems and experiential perspectives and reviews materials concerning the situations of minority groups living within dominant cultures.

The Canadian Virtual Hospice (virtualhospice.ca), an online palliative care (PC) knowledge translation resource, recognized the need for a tool to empower patients and families to access culturally-sensitive EoL care. In order to support the development of this tool, it is important to understand the attitudes, behaviours and utilization patterns of EoL care by culturally- and spiritually-diverse groups and associated gaps in practice, delivery of healthcare services and research. This paper presents a comprehensive scoping review exploring barriers and enablers encountered by cultural and spiritual groups when accessing EoL care at the individual, community and systems level.

## Methods

Scoping reviews systematically assess the breadth of a body of literature in a particular research area [10, 11]. Scoping reviews are particularly useful when investigating nascent and/or abstract concepts in order to map key themes in a research area and help to identify gaps in existing literature [12, 13]. The scoping review method was selected because, at present, little is known about culturally- and spiritually-sensitive interventions for EoL care. Using this methodology, we reviewed, categorized and synthesized a large volume of peer-reviewed and grey literature in which our conceptualization of cultural-sensitivity centers on the consideration of individual needs and preferences based on their unique identity and positionality (associated with ethnicity, gender, age, social class, acculturation); and according to Sperry “the capacity to anticipate likely consequences of a particular cultural problem or issue and respond to it empathetically” (p. 412–413). Likewise, spiritual-sensitivity emphasizes spiritual and religious awareness and empathy when responding to an individual’s faith-based concerns [14, 15].

Predefined search terms (see Table 1), covering academic, specialized and grey literature, were selected based on a comprehensive coverage of the four following underpinning notions: palliative care, culture, spirituality and strategy. For example, terms such as palliative were included but not dying as our research question surrounds the concept of end of life care and not specific experiences of dying per se. These terms were used to capture relevant peer-reviewed publications and grey resources across a range of disciplines of interest (i.e., humanities, social sciences, medicine, gerontology, nursing, policy and psychology).

**Table 1:** Search terms used in electronic databases and search engines

Search terms	
Palliative care	‘End of Life,’ ‘Palliative,’ ‘Care*,’ ‘Advanced,’ ‘Terminal,’ ‘Illness’
Culture	‘Cultur*,’ ‘Ethni*,’ ‘Divers*’
Spirituality	‘Religio*,’ ‘Spiritu*,’ ‘Faith*’
Strategy	‘Intervention*,’ ‘Video*’

Note: The asterisk next to search terms was used to retrieve distinct variations of the root word

A comprehensive review of five databases and nine websites (see Table 2) was conducted between June–August 2014 operationalized by the identification of:

**Table 2:** Complete list of electronic sources searched for the scoping review

Databases, search engines and content-relevant websites	N
Academic	5
PsychINFO	
CINAHL	
Web of Science	
ATLA Religion Database	
AgeLine	
Grey (including Government and Non-Governmental Organizations)	9
Google	
Open Grey – System for Information on Grey Literature in Europe	
Canadian Cancer Society	
Canadian Psychosocial Oncology Partners	
Growth House	
American Hospice Foundation	
Lien Foundation	
Centre for Advanced Palliative Care	
Marie Curie Organization	
Total	14

i. Reports and other print and digital material analyzing or documenting the degree to which specialized support is needed and available to cultural and or faith-based populations upon diagnosis of a life-limiting or advanced illness
ii. Supports, including but not limited to materials and programs designed specifically to facilitate cultural and spiritual populations access quality EoL care in health systems

Electronic search sources were selected to ensure: a comprehensive social science source to encompassing a wide disciplinary range; the inclusion of health and religious perspectives; as well as aging related issues given that end of life is strongly associated with older ages.

Guided by the inclusion/exclusion criteria (see Table 3), titles and abstracts were assessed to evaluate the articles for relevance followed by a scan of duplicate articles, which resulted in a final subset of 117 articles (*N* = 93 peer-reviewed publications; *N* = 24 grey resources).

**Table 3:** Inclusion and exclusion criteria

Inclusion	Exclusion
Published/created between 2004–2014 Focuses on cultural or spiritual diversity Focuses on end-of-life/palliative care Available free-of-charge or are available through university library services Written/created in English	Published/created before 2004 Not focused on cultural OR spiritual diversity AND end-of-life/palliative care Require a fee OR not available through university library services Resources in languages other than English

Note: No restrictions were made on methodological design or geographical location

Article content in both peer-reviewed and grey resources were extracted and coded into a spreadsheet for the full-text review. Inter-rater reliability was then undertaken. Kappa statistic was calculated for a random sample of 25 % of the records that were rated independently by each researcher. The inter-rater agreement for the full-text review was 0.965. Any discrepancies between the reviewers’ ratings were discussed until a consensus was reached.

A data extraction form was created with specific codes to guide the extraction of details of selected papers. The extraction form was tested by the researchers (MF, JS) to ensure consistency. During this process, the researchers made notes on which codes captured information relevant to the study and which additional codes were needed. The research team agreed that records included for charting could still be excluded during the data extraction process, for example, if the researcher found that a study did not pertain to the goals of the scoping review and/or did not fit the inclusion criteria then that article was excluded. Alongside data extraction, an annotated bibliography was created and abstracts of the final subset were analyzed thematically [16]. Since there was substantial heterogeneity among included studies, the majority of the data were synthesized descriptively. Quality assessment was not conducted as it is not a required element of a scoping review [10, 12].

**Fig. 1:**
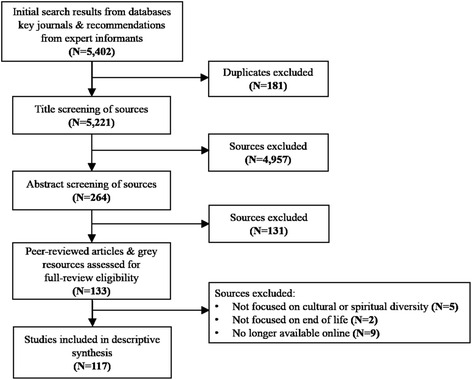
Scoping review search strategy

The search strategy, emerging themes, decision-making and findings were reviewed and validated by other members of the research team (GH, SS) as an additional measure of rigor. Figure 1 provides the breakdown of search results according to the different phases of the scoping review search strategy.

## Results and discussion

### A thematic analysis of findings

Eight themes emerged from the data. These were: (i) the need for culturally- and spiritually-sensitive PC; (ii) the impact of spiritual support on quality-of-life of terminally ill people; (iii) the role of families in EoL decision-making; (iv) cultural factors impacting EoL decision-making; (v) EoL preferences by ethnicity and gender; (vi) the diverse needs of hospitalized patients and implications for clinical practice; (vii) cultural competence and providers’ values impacting healthcare decision-making and (viii) interventions to inform and facilitate culturally-sensitive EoL care.

#### Theme 1: the need for culturally- and spiritually-sensitive palliative care

Over half of the reviewed studies (*N* = 49) originated in the United States (US). These provided evidence for cultural and age-related differences in EoL care, particularly, (i) help seeking behaviours, (ii) healthcare access and (iii) pain and symptom management preferences [17, 18].

Substantial disparities in advance care planning exist between ethnic minority groups and persons from Western cultures [19, 20]. Potential factors explaining these disparities include different conceptualizations of PC; varied beliefs and attitudes between HCPs and ethnic minority patients; socioeconomic status; lack of healthcare coverage, gendered beliefs and responsibilities, [17] and historical contexts shaped by colonialism that specifically impacted health beliefs and understandings of Aboriginal peoples [21].

Culturally- and spiritually-appropriate interventions have promoted dialogue regarding EoL care and treatment preferences among older ethnic minority adults, family members and healthcare professionals [22, 23]. Exploring diverse approaches to EoL can help enhance meanings and understandings of life and death in both Western and Eastern societies [24]. For example, a needs assessment of people living with end-stage renal disease in Thailand resulted in the development of a home-based PC model which allowed HCPs to better respond to the unique cultural and spiritual requirements of Thai people through integrating Buddhist spiritual practices [25].

While there was consensus within the literature that religious issues and meanings of holistic care (including spiritual practices and beliefs) should be openly discussed with patients and families, it was recognized that this may not always be feasible or appropriate in clinical practice [24]. For example, the Canadian fee-for-service model of healthcare delivery prevents in-depth spiritual and religious EoL discussions between patients and family doctors as it makes no provision for billing for such supports [26]. Responsive EoL care has been demonstrated as a universal need; yet these structural barriers create the potential for unwitting discrimination that results from limited in-depth discussions of an individual’s faith-based practices [27]. Despite the need to integrate culturally- and spiritually-sensitive PC into mainstream healthcare practice, structural barriers such as the remuneration process for doctors in Canada may prevent holistic care.

It is clear that many religious values and principles that inform PC are similar within and between groups. Nevertheless, we need to be cognizant that interventions for EoL need to be culturally-tailored [28]. According to Cheraghi [29], religions worldwide play an important role in shaping cultural understandings of life-and-death and to live a meaningful life. Diverse religious beliefs and practices, including rituals should inform the provision of EoL care by HCPs. For instance, in Muslim cultures, it is believed that death is not only the decline of our physical form and biological processes, there is also a strong belief that the spirit continues beyond death; and that dying is a transitional pathway towards the next life. Sacred texts (the Quran, Bible, etc.) are important sources of influence that inform adherents dying experience and enhance peace of mind [29, 30]. Acknowledging and developing a basic understanding of EoL beliefs from various faith groups needs to be better integrated into Western PC models [29].

Current research reveals that minority groups are less likely to utilize PC services in western societies in comparison to the mainstream culture. This is often due to a lack of knowledge about hospices or PC practices, a greater focus on family-centered decision-making models within these cultures, and preferences for less aggressive EoL care within these communities. In terms of acquiring new knowledge, existing research on EoL care approaches are often driven by post-positivist approaches that fail to incorporate the perspectives of cultural communities. In contrast, qualitative methods (such as ethnography, participatory action research and phenomenology) have been particularly effective in generating culturally-sensitive knowledge on EoL care directly from ethnic minority care-providers, family members and patients [31]. However, the limited application of qualitative methodologies hampers our understandings of EoL care enablers and barriers by ethnocultural groups [31].

It has been recommended that future research should: explore theoretical and causal mechanisms underpinning disparities in EoL uptake; invest in longitudinal investigations; incorporate meanings and understandings of EoL care from diverse patient groups and settings; adopt diverse and rigorous methodological approaches; and develop interdisciplinary culturally sensitive interventions [32]. Existing interventions addressing issues associated with culturally-sensitive PC have been criticized as a ‘cookbook’ approach [31]. These may inadvertently contribute to further generalizations, inaccuracies and contribute to misunderstandings of EoL practices, beliefs and rituals within these groups [31]. This process of ‘essentializing’ may contribute to further stereotypes or myths about ethnic minority groups. For example, while individuals within these religious groups likely share a set of common beliefs, it is recognized that within these groups there is individual and situational variance in beliefs. [31].

A common theme that emerged from several studies is the need for engaging patients and family members in dialogue when communicating EoL decisions between the patient and HCPs [31]. Generally, there is a need for intercollaboration, community cooperation, experiential knowledge synthesis in order to drive effective EoL care for ethnocultural groups [33]. This should also involve mobilization initiatives, public agencies and grassroots organizations as well as HCPs from public and community sectors [33].

#### Theme 2: impact of spiritual support on quality of life of terminally Ill people

Research highlights the importance of spiritual, religious and personal beliefs on individuals’ sense of inner piece, hope and optimism, and quality-of-life [34]. Spiritual and religious beliefs and practices become more important and are expressed when a person is nearing death [35]. As a result, it is crucial that PC providers acknowledge and tailor their care to the diverse spiritual needs of their patients. Responding to these needs may be challenging, yet this process can be fulfilling for HCPs, patients and family members as this provides opportunities for mutual spiritual and religious growth through the exchange of new spiritual understandings and meanings [36]. The impact of having HCPs address spiritual and religious issues was shown to have a positive effect on increased hospice usage at the EoL, and less aggressive interventions and intensive care unit (ICU) admissions at the EoL. [37]. Conversely, a study of spiritual coping among cancer patients revealed that those who used spiritual coping mechanisms were more likely to prefer life sustaining treatments [38]. These findings identify the influential role that spiritual and religious beliefs play in EoL decision-making and the importance of having discussions that address the role and impact of spiritual and religious beliefs in EoL decision-making [38].

In recognizing diversity of beliefs and expression within the Muslim faith, Bloomer et al. [39] revealed a spectrum of beliefs ranging from ‘very traditional’ to ‘very liberal.’ Consequently, it is not possible nor is it practical to expect that HCPs have in-depth understandings of all Islamic and Muslim practices, especially given the cultural variations amongst different Muslim groups [39]. As such, a generalized ‘cookbook’ approach detailing traditional beliefs and practices of different cultures may actually create more harm for patients as not all beliefs and practices will be applicable due to differences that exist within cultural groups [39, 40]. Hence, an open and flexible approach, that acknowledges and respects individuality within the spectrum of beliefs and practices of various cultural and spiritual groups, is an increasingly important feature of culturally-sensitive EoL care [39]. In addition to taking a person-centred approach to cultural and spiritual issues [40], it is important for HCPs not become desensitized to the profound nature of death and dying and the ethical and medical decision-making issues that are prevalent across cultural and spiritual groups [41].

Another important part of spiritual care relates to the role that traditional or complimentary medicine plays at EoL among certain groups [42]. Within the Chinese culture, the teachings of Taoism, Confucianism, and Buddhism are coalesced with Chinese medicine, providing an alternate and at times contrasting approach to death and dying which may need to be addressed and potentially integrated into the care plan of members from these communities [42, 43]. Although there is general agreement within the Chinese culture that life and death are a natural part of the human lifespan, the possibility of ‘a good death’ is highly influenced by their religious, spiritual and philosophical beliefs [1]. A further example of the influence of cultural and spiritual beliefs on death and dying was reported in a study on Hmong people, who reflect a syncretic spiritually, combining animism with more formal faith traditions such as Christianity [44]. Many Hmong persons believe that while their souls continue along a path towards the unseen world, the nature of this journey is influenced by bereft family members participation in funeral rituals [44].

The perceptions of a ‘good death’ amongst African-Americans is shaped strongly by spiritual beliefs and practices, and the role that God plays in determining their physical and spiritual health [45]. God’s influential role in the health of African-Americans is further illustrated in the belief that the HCP is God’s instrument of healing [45]. As such, many African Americans reject physician-assisted suicide and advanced directives limiting life-sustaining treatments on the grounds that they are counter to God’s will, reflect a lack of faith, and diminish the possibility of a divine intervention or miracle [46, 47]. Due to the important role of spiritual beliefs within the African-American community, it is important for HCPs to understand the pivotal role that spiritual beliefs play in coping and EoL decision-making [47].

Clearly, issues surround EoL decision-making and care delivery are strongly influenced by spirituality, religion and culture as well as the beliefs of individuals, families, communities and other social structures within these groups [48]. The application and transferability of the principles of Western bioethics within these cultures has been criticized for failing to appreciate the role and influence that these cultural and religious beliefs have on EoL decision-making [48, 49]. Consequently, inclusive and holistic approaches (which include the incorporation of diverse spiritual and religious beliefs, values, practices and expectations for care) are crucial for enabling patients to articulate complex preferences that are often suppressed within the context of healthcare provision [50]. It is important for persons that are approaching death to feel confident that they can share the spiritual and religious beliefs which shape their EoL wishes, the type of care that they receive and ultimately the achievement of ‘a good death’ [50].

#### Theme 3: role of families in end-of-life decision-making

The role of family in EoL care and decision-making are crucial within different cultural contexts as they help shape the experience of caregiving and dying. It is well documented that the engagement of family caregivers when providing spiritual and religious care within PC contexts is underutilized. This review highlights how familial understandings of concepts such as health and disease are based on personal experience and cultural factors such as language, family values and faith [7]. According to Edwards et al. [8], familial relationships form an integral part of meeting spiritual needs, particularly when patients are spiritually distressed when dealing with their terminal condition. Barriers to spiritual care (within the context of healthcare provision) extend from individual (e.g. personal preferences and time pressure) to macro level factors (e.g. institutional ethos and providers’ lack of cultural knowledge) [8]. HCPs must consider the varying cultural identities of family caregivers and the role that they play within the context of the dying process in order to provide more sensitive care [51]. The literature concludes that developing service provision that matches unique individual needs, crucially involves family members in the EoL care process [7].

Research reveals that family members from cultures that revere filial piety have significant influence on the dying wishes of the individual in terms of treatment options as well as religious and spiritual needs [1, 9]. For example, within Chinese cultures, Confucianism and filial piety are values which are integral to family structures and relationships [52]. Filial piety requires the younger generation to honour their elders’ wishes regardless of difficult social and economic circumstances, noting that contravening this can bring shame on the family. However, the pressures imposed by filial piety on the younger generation can create a set of uncertainties concerning: (i) whether children want to make decisions at this difficult time for their parents (ii) whether the parents want their children to make those decisions and (iii) where should responsibility reside (i.e. the eldest son or all of the children). As a result, some children resist adherence to the principles of filial piety; and in the context of EoL care, may not ensure that parental decision-making and wishes carried through [53]. Furthermore, in a study of Thai Buddhist families, a family member’s dying experience was not viewed as a time of suffering but rather an opportunity to share time together, achieving a peaceful death in the process [54].

#### Theme 4: cultural factors impacting end-of-life decision-making

In general, there were a number of cultural factors that influenced EoL care decision-making and completion of an advance directive. These included: incomplete life tasks; hope and hopelessness; acceptance and preparation; perspectives on suffering and death and dying; social support networks; barriers to accessing EoL care and general mistrust of the healthcare system [55, 56]. In one study of African-American patients, it was reported that mistrust and suspicion of health professionals and the healthcare system created a substantial barrier to accessing EoL care [55].

In a separate study of African-Americans, EoL decision-making was influenced by the desire to maintain everyday activities, personal autonomy (specifically, maintaining control of personal healthcare), and preventing the overburdening of caregivers and family members [46]. Another study found that resistance to advance care planning for African Americans stems from the belief that God controls the timing and nature of death [57].

Cultural influences were also identified in a study of dying patients in Hong Kong, many of whom desire to die at home [58]. Only 2.7 % of patients died at home, however, largely due to the influence of social and culture barriers including limited housing, crowded living environments, and perceived threat of devaluing real estate property should a death occur at home [58]. The same study identified social and cultural taboos (e.g. idea that speaking about death may bring upon death) has significant influencers on perceptions of ‘a good death’ [58].

#### Theme 5: end-of-life preferences by ethnicity and gender

Decisions concerning EoL care were also influenced by ethnicity and gender. For instance, it was found that preferences for communicating about terminal illnesses differed between cultural groups (White Americans and Americans of Arab descent) and was compounded by different requirements of each gender [59]. This study revealed that EoL wishes for some Americans of Arab descent included making peace with others as an important EoL issue [59]. Within the same sample, it was reported was that the majority of participants were against assisted suicide, prolonging life through artificial methods, spending their last days in nursing homes and disclosing bad news to patients who are nearing EoL [59]. In a separate study of Hispanic and African American, women were more adverse to physician assisted suicide in comparison to men from the same cultures [59]. The influence of age, was identified as a factor in EoL decision-making in another study, as older Hispanic persons preferred comfort care and less aggressive treatment than younger persons from the same culture [60]. Despite having these preferences, research suggests that only a few communicated this to their HCPs and family members [60]. This disparity placed some older Hispanic people at risk for receiving highly aggressive EoL treatment and care [60].

Research has also highlighted that individuals from minority backgrounds are more likely to experience difficulty communicating their concerns and preferences relating to EoL to their HCPs [61]. In addition to variance between groups, Duffy et al. [61] identified differences between women and men within these groups in relation to perceived discrimination at the EoL. A study of Muslim women identified a number of cultural barriers (e.g. withholding ‘bad’ information from family members against wishes of HCPs) associated with EoL care, whilst African-Americans believed that their cultural background was a factor in the EoL care they received and Caucasian groups were more likely to emphasize age as a discriminatory factor [61]. A separate study identified the importance Caucasian groups placed on having different options and choices to meet their EoL care needs, while it was found that African-Americans were less likely to consider admission to a nursing home as a care option [59]. Findings from a similar study illustrated that some African-American patients tended to prefer using life-sustaining interventions toward the EoL, although their cultural background did not appear to impact decisions to refuse or withdraw some variations of life-sustaining technology, with spiritual beliefs playing an important in these decisions regardless of religiosity [62].

Another important finding challenges anti-essentialist views by disregarding the heterogeneity of African cultures and traditions [63]. Different African groups can have very different needs at EoL and these needs to be taken into account when delivering EoL care. For example, for Xhosa people from East Africa, the facilitation of a good death is equally as important as the traditional rituals and practices post death. Pre- and post- death practices include: relief of psychosocial suffering; speaking to family members at the deathbed; and engagement with spirituality at the EoL [64]. Within different African traditions, while there are similarities across rituals when someone dies these could vary according to traditional family values and customs or by the dying person’s wishes [64]. Future research should consider the heterogeneity that exists between and within ethnocultural groups.

With respect to preferences surrounding advanced directives, findings from one study revealed that, in general, minority groups lacked sufficient knowledge and are therefore less likely than Caucasian groups to engage with advanced directives, with African-Americans being likely to prefer use of life support [65]. Meanwhile, some Hispanics and Asian persons indicated preference toward family centered decision-making processes, which was not a prominent preference in other ethnic groups [65]. Lastly, for many groups from South and East Asian cultures, preference to die at home was strongly influenced by their need to perform various cultural and religious rituals not easily transferrable to hospital settings, with the authors noting that achieving ‘a good death’ in these cultures requires accommodation to EoL spiritual practices [66].

#### Theme 6: diverse needs of hospitalized patients and considerations for practitioners

In caring for diverse patients facing EoL, a number of recommendations for HCPs emerged from our review including the need to: actively engage family members; incorporate cultural and spiritual values into care; negotiate with family members the care plan in accordance with the patient’s changing health status; and improve cultural and appropriate language communication [67, 68]. Factors that influence care for various ethnic minority groups, in general, include communication barriers, differences between Western and traditional foods, and differing cultural beliefs and customs [69]. For example, it has been reported that assessing the level of pain of some ethnic minority patients is difficult since the concept of pain is subjective and influenced by a combination of social, cultural and spiritual factors [70]. Thus, accounting for various social, cultural and spiritual factors in conducting pain assessments is crucial, as inaccurate determinations of pain by HCPs can result in the patient receiving inadequate pain medication [70]. Furthermore, it was reported that there are also gaps in understandings for the availability of culturally- and spiritually-diverse resources at the EoL such as sacred texts, prayer rugs in Muslim cultures, and the influence of rabbis and religious beliefs in relation to medical procedures in the Orthodox Jewish tradition [71, 72]. Other needs include Muslim preferences for lowering their intake of sedatives at the EoL in order to recite prayers [71].

The literature also emphasized the importance of both educating cultural and religious groups about EoL care and the need to educate HCPs working at the EoL about cultural and spiritual beliefs within these groups [73]. This is particularly important as studies have reported that HCPs understandings of cultural variations in attitudes and values concerning EoL care have important practical implications that influence individual decision-making towards medical decisions as well as EoL preparation and needs [74, 75]. For example, the EoL needs identified for Indian groups vary according to the beliefs and values they associate with their social and community statuses; gender; and dialect [74, 75]. While our review synthesized the disparate literature on the different needs of culturally- and spiritually-diverse hospitalized patients, it also confirmed that addressing such requirements is a vastly complex endeavor, particularly for HCPs who are challenged in balancing religious and cultural beliefs and values with the beliefs and values of mainstream healthcare.

#### Theme 7: cultural competence and providers’ values in healthcare decision-making

HCPs’ insufficient cultural and spiritual knowledge and understandings of persons from diverse backgrounds is cited as one of the primary reasons why ethnic minority groups access EoL care less often then white European groups [76]. Reasons behind this low uptake of EoL care revolve around insufficient cultural competence training and lack of representation of ethnic, cultural and spiritual diversity among health service providers [77].

Healthcare practitioners reportedly felt ill-prepared to engage in faith-based discussions especially about religious and spiritual beliefs, practices and values that were different to their own [78]. For instance, a study in the UK found that, in general, PC providers were more likely to be Christian and of white-European descent and lacked knowledge of other religions and spiritualties [79]. Meanwhile, HCPs positioned as non-religious more often reported: utilizing continuous deep sedation until death; considering patients’ decisions to end life; and having had conversations about such decisions with patients deemed capable of participating these discussions [79]. The level of acculturation of primary caregivers of ethnically diverse patients was shown to also impact differences in EoL preferences and medical decision-making [80].

Clinical EoL encounters between HCPs and service users are influenced by religious and spiritual identities [81]. Religious and/or spiritual identities do not occur in isolation from other social identifiers such as gender, ethnicity and class [81]. For example, an individual’s spirituality is often negotiated through gendered and/or cultural norms, roles and responsibilities, or in the case of socioeconomic status, persons perceived to be of lower socioeconomic status were associated with having questionable morality and seen as being less spiritual [81]. Locating spirituality as a point of connection between the provider and patient was recommended as a means of creating safe spaces for open communication [81, 82]. Lack of communication efforts by service providers may lead to poor outcomes for persons approaching EoL [83]. For example, insufficient communication efforts between providers and patients was reported to influence decisions for do not resuscitate orders; reduce access to life-sustaining therapies; limit meaningful discussions with patients on EoL decision-making, ultimately create contexts of inadequate PC for dying persons [83].

The appreciation of culturally- and spiritually-diverse beliefs, practices, values and traditions benefited HCPs, particularly through building better rapport with individuals and family members, which lead to improved EoL service uptake [84]. Providers who had encountered people of diverse cultures and those exposed to organizational approaches to culturally- and spiritually-sensitive care provided more effective EoL care [85]. This finding demonstrates that effective EoL care depends to some extent on HCPs previous experience and active efforts to understand their patients’ cultural and spiritual practices, beliefs, values and traditions [86]. Perhaps one of the primary challenges for HCPs is the ability to reflect and problematize their own unacknowledged anxieties, prejudices, biases and fears about other cultural and spiritual beliefs, practices and values that are different to their own [87, 88].

#### Theme 8: interventions to inform and facilitate culturally-sensitive end-of-life care

Of the 116 peer-reviewed articles and grey resources included for full-text review, 33 concerned interventions aimed at improving culturally-sensitive EoL care. Thirteen interventions focused on education and training for students and HCPs in medicine and nursing [43, 89–100]. Educational programs to improve cultural awareness and sensitivity of students across health professions have combined online learning with interactive simulations emphasizing spiritual, cultural aspects of PC [89, 99]. Curriculums that integrate spirituality and culture with PC were noted as essential for improving the quality of care in Westernized health systems for multicultural and multi-religious communities [92, 94, 98]. Interprofessional educational programs that incorporated critical reflection sessions facilitated interprofessional dialogue and subsequently encouraged understandings of diverse needs [93]. Online [100–102]/multimedia features [103, 104], interactive dialogue [93, 105], self-reflection [93, 106, 107] and story-telling [108, 109] appear to be promising components for curriculums to improve cultural awareness and sensitivity and may be transferrable to other educational and healthcare settings.

Compared to modest in-person interventions, cultural competence scores were significantly higher for providers that had experienced specific educational cultural-competency training programs, particularly online training [90, 91]. For example, findings from one study found that web-based educational interventions to improve cross-cultural communication concerning EoL issues were useful for hospice workers as it introduced culturally-sensitive ways to assess situations and communication strategies with culturally- and spiritually-diverse groups at the EoL through online scenarios [91]. Online delivery methods were reported as convenient, user-friendly and interactive [91].

Other interventions aimed to improve understandings of and influence attitudes towards EoL care were through culturally-tailored material for family members and older persons approaching EoL [110–113]. Culturally-tailored print and online resources (e.g. in diverse languages) that presented knowledge about EoL care were effective for EoL planning and decision-making [110, 111, 114, 115].

Some educational material on EoL were delivered using relational in-person, peer-mentoring approaches and this method of delivery was deemed more effective than providing written material, primarily, due to the human component through the ability to build trust between the knowledge provider and knowledge user [111].

Lastly, seven frameworks and guides recommended for providing care for culturally- and spiritually-diverse persons approaching EoL and their family members explained the dynamic nature and the similarities between different cultural traditions as well as strategies for shared decision-making between providers and service users to overcome barriers at the patient-, system- and societal-levels [68, 116–121]. Two examples of such frameworks involve: (i) transcultural nursing concepts that aid hospice providers with assessments and interventions in multicultural situations [117] and (ii) strategies to bridge communication between terminally-ill patients and their family members with HCPs [119].

## Conclusions

Findings from this scoping review highlight a multitude of factors influencing the receipt of poor quality EoL care and subsequent experiences by culturally- and spiritually-diverse groups. Table 4 highlights key barriers related to each theme and future priorities for research and service development.

**Table 4:** Key barriers and future priorities for research and service development for end-of-life care

Themes	Barriers	Future priorities
The Need for Culturally- and Spiritually-sensitive Palliative Care	• Different conceptualizations of PC between ethnic minority groups and people from Western cultures • Varied EoL care attitudes and beliefs between HCPs, patients and families • Lack of fit of religious notions to medicalized health care provision	• Research into the development of tools (such as video explanations or online and print resources in multiple languages) to help ethnocultural groups understand the concept and availability of advanced care planning. • Research into the development of tools to support communication between HPCs and ethnocultural groups to discuss advance care planning • Research on how to facilitate open discussion between HPCs, patients and families on religious requirements within PC
Impact of Spiritual Support on Quality of Life of Terminally Ill People	• PC providers lack of understanding of the need for spiritual requirements and how to facilitate these within the context of PC • Decision-making in PC can follow too rigidly to the dominant cultural practices of the country where the PC is situated	• Research for the development of spiritual coping mechanisms that will enable PC providers to better understand and find ways to integrate the necessary spiritual practices into their care regime • Research to better understand how spiritual and religious beliefs impact EoL care decision-making of different groups to enable more inclusive and holistic approaches initiated within PC delivery
Role of Families in End-of-Life Decision-making	• The Western medical model often dominates practices of care making it difficult to appropriately integrate the voices of the patient and family members in EoL decision-making (particularly for those with divergent beliefs and practices) • Differences in EoL wishes, needs and requirements between family members, patients is often challenging for HCPs when providing care	• Research on how best to encourage cultural shifts from the biomedical perspective to more individual, person and family-centred approaches • Research on how HCPs can best negotiate different wishes/needs between family members and the patient and together develop a forward plan; knowledge gained can the this process can be integrated into current training for HCPs
Cultural Factors Impacting End-of-Life Decision-making	• General mistrust of the healthcare system due to lack of knowledge, particularly HCPs working practices when providing EoL care • Lack of knowledge on the types of EoL services available to patients and family members • Incomplete life tasks: patients do not feel ready to die	• Undertake research on the EoL working practices of HCP to create an information resource made available to patients and families • Undertake an environmental scan of available culturally-tailored services and resources, disseminate widely in multiple languages and ensure this is made available to patients and families and that it is kept up to date • Research on how best to counsel patients to come to a good resolution of incomplete life tasks to enable them to approach death in a prepared way
End-of-Life Preferences by Ethnicity and Gender	• Preferences for communication regarding terminal illnesses differed between cultural groups additionally were differentiated by gender • Patients perceptions of discriminatory attitudes from HCPs and this differed by gender • Essentialist views of diverse cultures and traditions • Differing place preferences for dying not feasible in clinical settings	• Undertake research on how to best inform HCPs on the heterogeneity that exists between and within ethnocultural groups • Research on preferences of ‘a good death’ by different ethnocultural groups
Diverse Needs of Hospitalized Patients and Considerations for Practitioners	• HCPs are challenged with balancing religious and cultural beliefs and values with the beliefs and values of mainstream healthcare • Communication barriers between HCPs and patients and family members • Differences between Western and traditional foods, • Differing cultural beliefs and customs i.e. about the concept of pain	• Develop processes and guidelines on how to best engage family members in the EoL care process • Research on how to best incorporate cultural and spiritual values into mainstream healthcare provision • Develop best practices and guidelines on how to best negotiate with family members the care plan in accordance with the patient’s changing health status • Research on developing resources to inform HCPs the diverse food preferences • Research on differentiated understandings of pain and pain care
Cultural Competence and Providers’ Values in Healthcare Decision-making	• HCPs’ insufficient cultural and spiritual knowledge and understandings of persons from diverse backgrounds • Insufficient cultural competence training and lack of representation of ethnic, cultural and spiritual diversity among health service providers • HCPs’ Inability to reflect and problematize their own unacknowledged anxieties, prejudices, biases and fears about other cultural and spiritual beliefs, practices and values	• Research on how to best locate spirituality as a point of connection between HCPs and patients to create safe spaces for open communication • Research on appreciation of culturally-/spiritually-diverse beliefs, practices, values and traditions • Develop resources on how HCPs can reflect and problematize their own unacknowledged anxieties, prejudices, biases and fears about other cultural and spiritual beliefs, practices and values
Interventions to Inform and Facilitate Culturally-Sensitive End-of-Life Care	• Insufficient funding mechanisms to build and create best practice resources for culturally-sensitive EoL care • Difficulties with dissemination and normalization within healthcare practice to enhance uptake of resources from HCPs	• Develop Interprofessional educational programs incorporating critical reflection and dialogue to encouraged understandings of diverse needs • Develop online/multimedia features, interactive dialogue, self-reflection and story-telling opportunities and resources to improve cultural awareness in educational and healthcare settings

Primary barriers to EoL care include cultural differences between HCPs, persons approaching EoL and family members; under-utilization of culturally-sensitive models designed for improved EoL care; language barriers; lack of awareness of cultural and spiritual diversity issues; exclusion of families in the decision-making process; personal, racial and religious discrimination and lack of culturally-tailored EoL information to facilitate decision-making and uptake of care for culturally- and spiritually-diverse communities. Indeed, this may be reflective of the neglect of cultural factors within current models of care provision. Selman et al. [122] has suggested that more holistic models of care are required, integrating the experience of ill health and conceptualizations of the meaning of EoL care which are intrinsically imbued with cultural and spiritual meaning.

Enablers of culturally- and spiritually holistic care identified in the literature stressed the need for active efforts to: engage family members; incorporate diverse cultural and spiritual values; negotiate with family members the changing needs of the patient to both maintain and release control of the individual’s EoL care and improve as well as sustain culturally- and linguistically-effective communication between HCPs and service users. A key challenge for HCPs is the ability to reflect on and problematize their own unacknowledged anxieties, prejudices, biases and fears about other cultural and spiritual practices, beliefs and values that are different to their own. This suggests that engagement in self-reflection and reflexive development of understandings concerning the intersections between care provision, cultural and spiritual meaning at EoL should enable deeper understanding and empathy towards persons of different cultural and spiritual backgrounds. Such reflexivity should progress beyond personal care tasks and into the structure of services as well as the process of care delivery [122].

This scoping review has shown that much of EoL care research has specifically focused on EoL decision-making while everyday service experiences have been relatively neglected. Without knowledge of such experiences, it is difficult to ensure that EoL care is delivered in accordance with cultural and spiritual expectations, leaving service users unsatisfied. Our scoping results are also indicative of the fact that there has been little movement toward developing interventions for promoting culturally- and spiritually-sensitive EoL care. Interventions identified were largely educational in nature, aimed at students in medicine and nursing and other HCPs. While educational interventions are valuable, practice-based interventions were not in evidence; creating a gap in knowledge regarding good practice in home, hospital and hospice settings. Despite the lack of rigorous intervention evidence on what constitutes best practice in cultural and spiritual EoL care, there is a need for guidelines and recommendations and quality fireworks to evaluate effectiveness in cultural and spiritual care practices. Such guidelines and recommendations can proceed from our academic understandings as a first step towards improved and more equitable practices, especially where they promote a strong person and family focused lens which locates culturally and spiritual EoL care as a process rather than a mechanistic exercise, a process which engages with the experiences of all persons in receipt of care.

In terms of study limitations, although we identified and synthesized a substantial volume of literature to address critical knowledge gaps associated with culturally- and spiritually-sensitive EoL, this review is not without weaknesses; as such, findings and suggestions for improved EoL care should be interpreted with some caution. Firstly, our inclusion criteria may have been too broad in scope, which could have contributed to the high quantity and increased heterogeneity of results during the earlier stages of article selection process, namely the title and abstract screening phases. Secondly, only English articles were included and as a result there may be relevant literature on this topic published in other languages. Lastly, a substantial proportion of the studies included in this report were based in the US. US studies can provide important direction for high quality culturally- and spiritually-sensitive care in other national settings, however it is important to understand the enablers and barriers to EoL care within a Canadian context if empowerment tools for Canadians are to be developed.

### Ethics approval and consent to participate

Not applicable.

### Consent for publication

Not applicable.

### Availability of data and materials

The raw data set is made available as an additional supporting file of this manuscript. Please see link below to Additional file 1.

## Additional file

Additional file 1: A body of data consisting of key features, characteristics and summaries of all included studies. The file is entitled, “CS_EoL_Data_Charting_Form_12Jan2015_FINAL”. (XLS 605 kb)

## Abbreviations

EoL: end of life
HCPs: healthcare providers
ICU: intensive care unit
PC: palliative care
US: United States
